# Supplementary material for: The Impact of Cold Ischaemia Time on Outcomes of Living Donor Kidney Transplantation: A Systematic Review and Meta-Analysis
Source: J Clin Med. 2022 Mar 15;11(6):1620. doi: 10.3390/jcm11061620 (PMC8951281; doi:10.3390/jcm11061620)
Supplement: Supplementary file 1 [file jcm-11-01620-s001.zip › jcm-1593401-supplementary .pdf]

Table S1. Risk of bias assessment of included studies in the meta-analysis. Overall score was moderate in case assessment on all levels was low or moderate. To be scored as low all sections needed to be low and this is comparable with an RCT, for serious risk of bias at least one assessment should be classified as serious.

Study ID								
	Bias due to confounding	Bias due to selection of participants	Bias due to measurement of outcomes or intervention	Bias due to deviation from the intended intervention	Bias due to missing data	Bias due to measurement of outcomes	Bias due to selection of reported results	**Overall risk of bias score**
**Gill** (2017)	Moderate	Low	Low	Low	Moderate	Low	Low	**Moderate**
**Krishnan** (2016)	Moderate	Low	Low	Low	Low	Low	Moderate	**Moderate**
**Nassiri** (2019)	Moderate	Low	Low	Low	Moderate	Low	Moderate	**Moderate**
**Nath** (2016)	Moderate	Low	Low	Low	Low	Low	Low	**Moderate**
**Redfield** (2016)	Moderate	Low	Low	Low	Moderate	Moderate	Low	**Moderate**
**Roodnat** (2003)	Moderate	Low	Low	Low	Low	Low	Low	**Moderate**
**Segev** (2011)	Low	Moderate	Moderate	Low	Low	Moderate	Low	**Moderate**
**Simpkins** (2006)	Moderate	Low	Low	Low	Moderate	Low	Low	**Moderate**

Table S2. sensitivity analysis. Explanation: in the first column, the study outcome for which the meta-analysis is performed is described together with the corresponding statistical description. In the table next to it, you can find the sensitivity analysis. Behind each author are the columns heterogeneity, I^2 and overall p-value. These describe what happens to these values when the study is removed from the meta-analysis. Abbreviations: LS = less significant, MS = more significant, NA = not applicable, NS = non-significant, SD = slight decrease (a decrease that does not lead to a change in significance), SI = slight increase (that does not lead to a change in significance), / = no remarkable change.

Outcome measure and analysis	Author	Heterogeneity	I2 (%)	Overall P value
**DGF CIT < 4 vs. CIT >4**				
Heterogeneity: $Tau^2 = 0.04$; $Chi^2 = 14.34$, df = 4 (P = 0.006); $I^2 = 72\%$				
Test for overall effect: Z = 4.27 (P < 0.0001)		**0.006**	**72%**	**< 0.0001**
	**Gill**	not significant	SD	MS
	**Krishnan**	/	/	/
	**Nassiri**	/	/	/
	**Nath**	/	/	/
	**Simkins**	/	/	/
**Graft survival 1year DS CIT <4 vs. >4h**				
Heterogeneity: $Tau^2 = 0.00$; $Chi^2 = 0.82$, df = 1 (P = 0.37); $I^2 = 0\%$				
Test for overall effect: Z = 3.40 (P = 0.0007)		**0.37**	**0%**	**0.0007**
	**Nath**	NA	NA	/
	**Simpkins**	NA	NA	NS
**Graft survival 5year DS CIT <4 vs. >4h**				
Heterogeneity: $Tau^2 = 0.00$; $Chi^2 = 1.24$, df = 3 (P = 0.74); $I^2 = 0\%$				
Test for overall effect: Z = 2.10 (P = 0.04)		**0.74**	**0%**	**0.04**
	**Krishnan**	/	/	/

	**Nassiri**	/	/	/
	**Nath**	/	/	NS
	**Simkins**	/	/	/
**Acute Rejection**				
Heterogeneity: $Tau^2 = 0.06$; $Chi^2 = 16.35$, df = 2 (P = 0.0003); $I^2 = 88\%$				
Test for overall effect: Z = 1.01 (P = 0.31)		**0.0003**	**88**	**0.31**
	**Krishnan**	/	/	SI
	**Nath**	not significant	SI	significant
	**Simpkins**	/	/	SI
**Patiënt survival 1 year <4 vs >4**				
Heterogeneity: $Tau^2 = 0.01$; $Chi^2 = 1.19$, df = 1 (P = 0.28); $I^2 = 16\%$				
Test for overall effect: Z = 1.59 (P = 0.11)		**0.28**	**16**	**0.11**
	**Krishnan**	NA	NA	SI
	**Nath**	NA	NA	significant
**Patient survival 5 year CIT <4 vs. CIT >4**				
Heterogeneity: $Tau^2 = 0.48$; $Chi^2 = 15.69$, df = 1 (P < 0.0001); $I^2 = 94\%$				
Test for overall effect: Z = 1.22 (P = 0.22)		**<0.0001**	**94**	**0.22**
	**Krishnan**	NA	NA	NS
	**Nath**	NA	NA	significant
**Graft survival 0 - 2-4h CIT**				
Heterogeneity: $Tau^2 = 0.00$; $Chi^2 = 2.05$, df = 3 (P = 0.56); $I^2 = 0\%$				
Test for overall effect: Z = 1.23 (P = 0.22)		**0.56**	**0**	**0.22**
	**Gill**	/	/	/

	**Krishnan**	/	/	/
	**Nath**	/	/	/
	**Simpkins**	/	/	SI
**Graft survival 0 - 4-8h CIT**				
Heterogeneity: $Tau^2 = 0.00$; $Chi^2 = 3.78$, df = 3 (P = 0.29); $I^2 = 21\%$				
Test for overall effect: Z = 2.77 (P = 0.006)		**0.29**	**21%**	**0.006**
	**Gill**	/	/	/
	**Krishnan**	SI	0%	/
	**Nath**	/	/	/
	**Simpkins**	/	/	SI
**DGF 0-2h vs. 2-4h CIT**				
Heterogeneity: $Tau^2 = 0.00$; $Chi^2 = 1.90$, df = 3 (P = 0.59); $I^2 = 0\%$				
Test for overall effect: Z = 2.57 (P = 0.01)		**0.59**	**0**	**0.01**
	**Gill**	/	/	NS
	**Krishnan**	/	/	/
	**Nath**	/	/	/
	**Simkins**	SI	/	MS
**DGF CIT 0-2h vs. 4-6h**				
Heterogeneity: $Tau^2 = 0.00$; $Chi^2 = 2.40$, df = 3 (P = 0.49); $I^2 = 0\%$				
Test for overall effect: Z = 8.77 (P < 0.00001)		**0.49**	**0%**	**0.00001**
	**Gill**	/	/	/
	**Krishnan**	/	/	/
	**Nath**	/	/	/
	**Simpkins**	/	SI	/
**DGF CIT 0-2h vs. 6-8h**				

Heterogeneity: Tau² = 0.00; Chi² = 0.11, df = 3 (P = 0.99); I² = 0%				
Test for overall effect: Z = 9.23 (P < 0.00001)		**0.99**	**0**	**0.00001**
	**Gill**	/	/	/
	**Krishnan**	/	/	/
	**Nath**	/	/	/
	**Simpkins**	/	/	/
**Graft survival 1 year CIT 0-2h vs. 2-4h**				
Heterogeneity: Tau² = 0.00; Chi² = 0.14, df = 1 (P = 0.70); I² = 0%				
Test for overall effect: Z = 1.72 (P = 0.09)		**0.70**	**0**	**0.09**
	**Nath**	NA	NA	/
	**Simpkins**	NA	NA	0.63
**Graft survival 1 year CIT 0-2h vs. 4-8h**				
Heterogeneity: Tau² = 0.00; Chi² = 0.46, df = 1 (P = 0.50); I² = 0%				
Test for overall effect: Z = 3.55 (P = 0.0004)		**0.50**	**0**	**0.0004**
	**Nath**	NA	NA	/
	**Simpkins**	NA	NA	NS
**Graft survival 5 year CIT 0-2h vs. 2-4h**				
Heterogeneity: Tau² = 0.00; Chi² = 1.78, df = 2 (P = 0.41); I² = 0%				
Test for overall effect: Z = 1.02 (P = 0.31)		**0.41**	**0%**	**0.31**
	**Krishnan**	SI	/	SD
	**Nath**	SD	SI	SI
	**Simpkins**	SD	SI	si

**Graft survival 5 year CIT 0-2h vs. 4-8h**				
Heterogeneity: $Tau^2 = 0.00$; $Chi^2 = 2.02$, df = 2 (P = 0.36); $I^2 = 1\%$				
Test for overall effect: Z = 2.56 (P = 0.01)		**0.36**	**1**	**0.01**
	**Krishnan**	/	si	/
	**Nath**	SD	si	si
	**Simpkins**	si	0	MS
**Graft survival 10 year CIT <4 vs. >4 hours**				
Heterogeneity: $Tau^2 = 0.03$; $Chi^2 = 3.01$, df = 1 (P = 0.08); $I^2 = 67\%$				
Test for overall effect: Z = 1.32 (P = 0.19)		0.08	67	0.19
	**Krishnan**	NA	NA	/
	**Simpkins**	NA	NA	significant

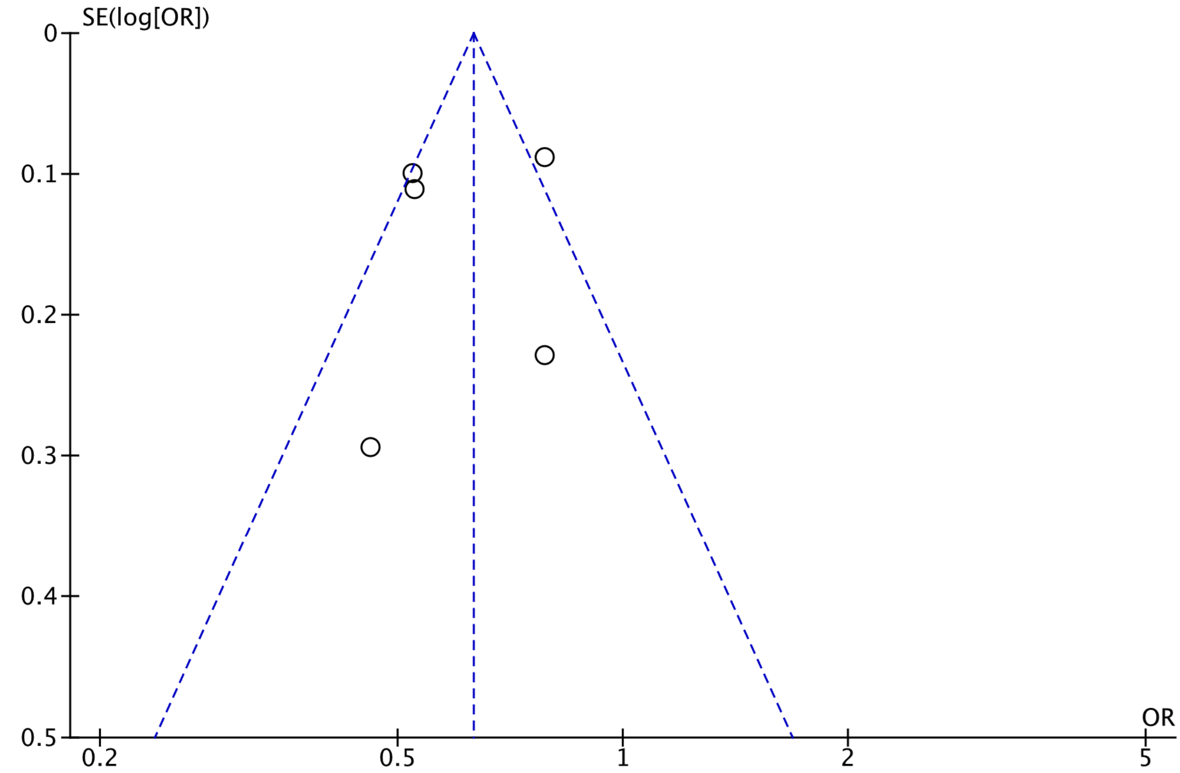

Figure S1. Funnel plot for DGF for CIT shorter and longer than 4 hours

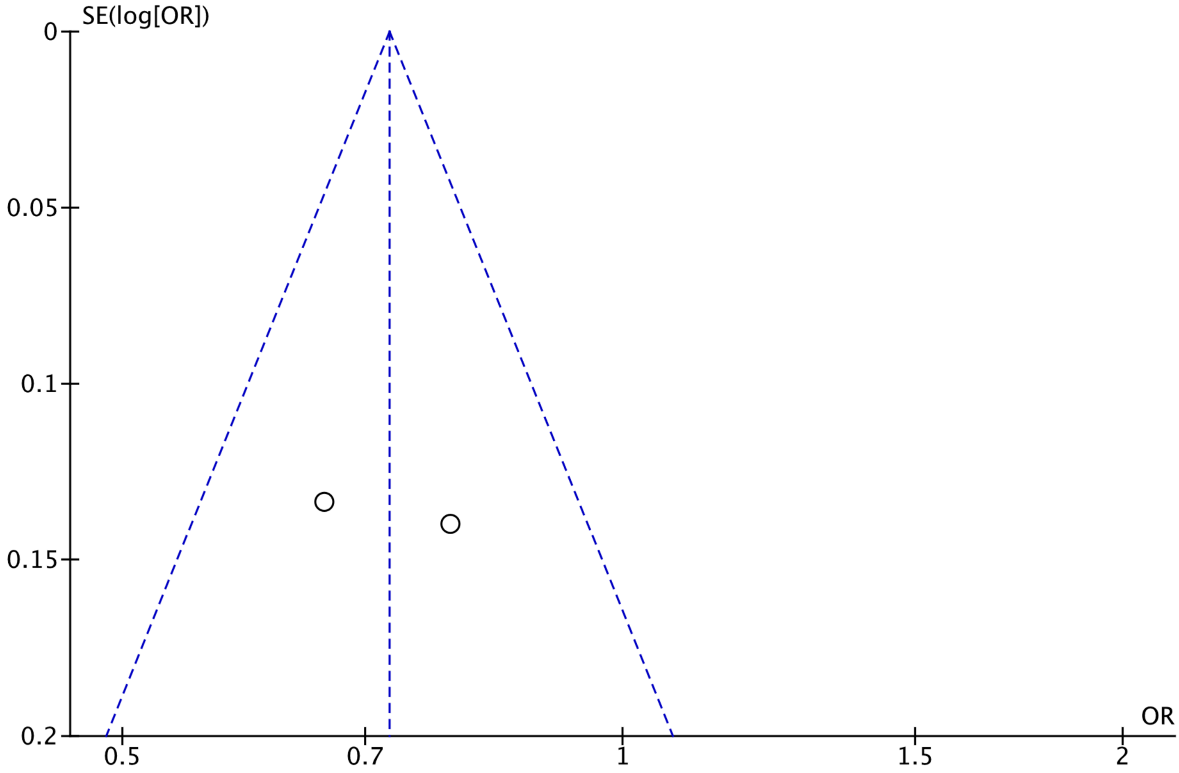

Figure S2. Funnel plot for 1-year graft survival for CIT shorter and longer than 4 hours.

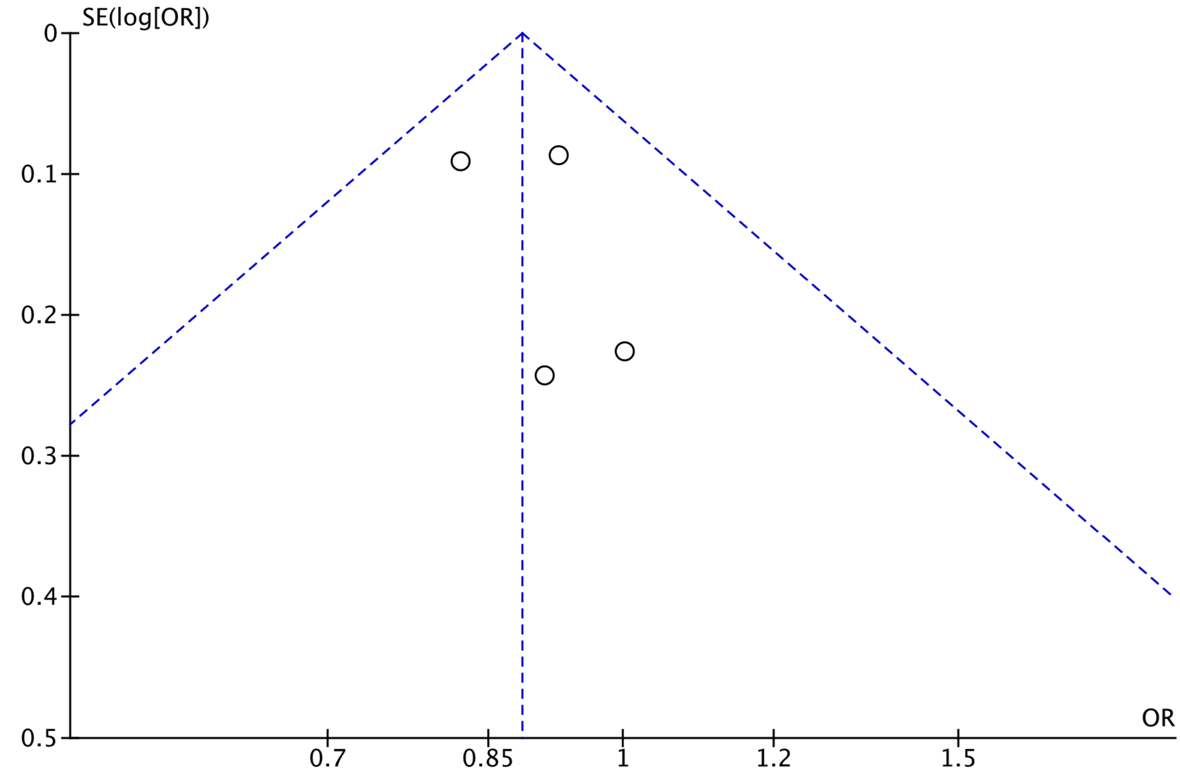

Figure S3. Funnel plot for 5-year graft survival for CIT shorter and longer than 4 hours.

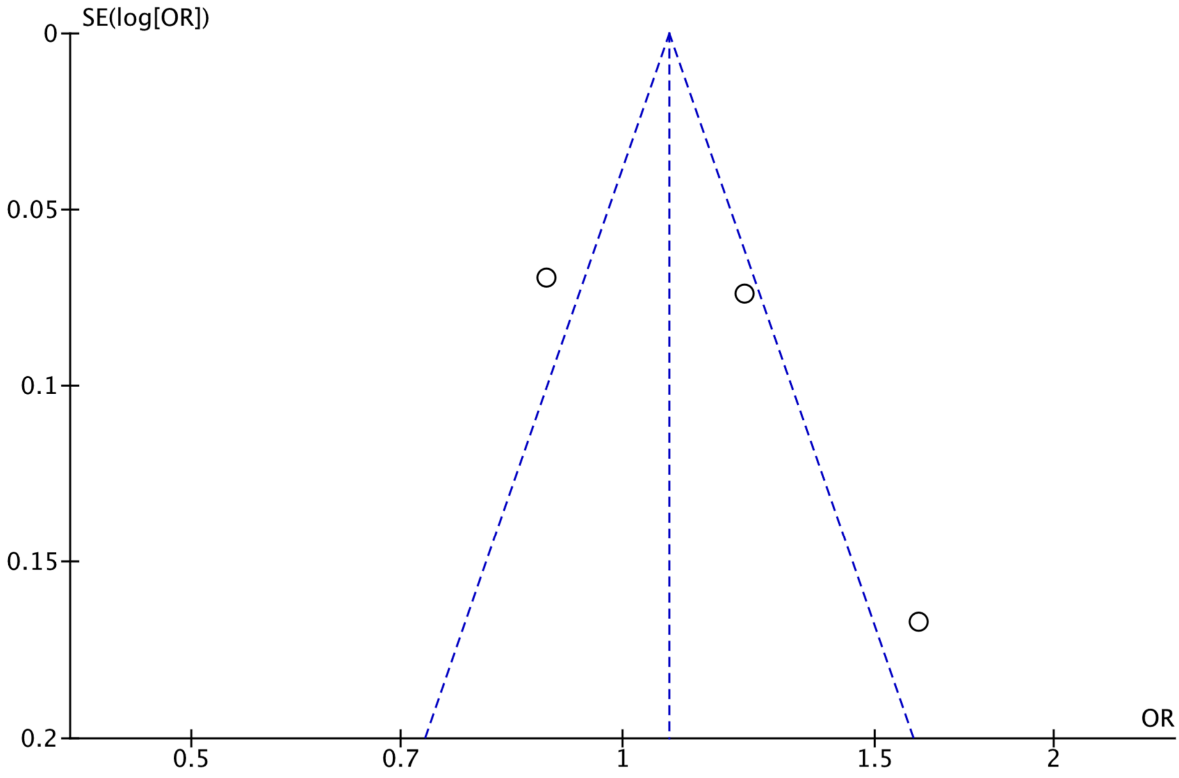

Figure S4. Funnel plot for acute rejection for CIT shorter and longer than 4 hours.

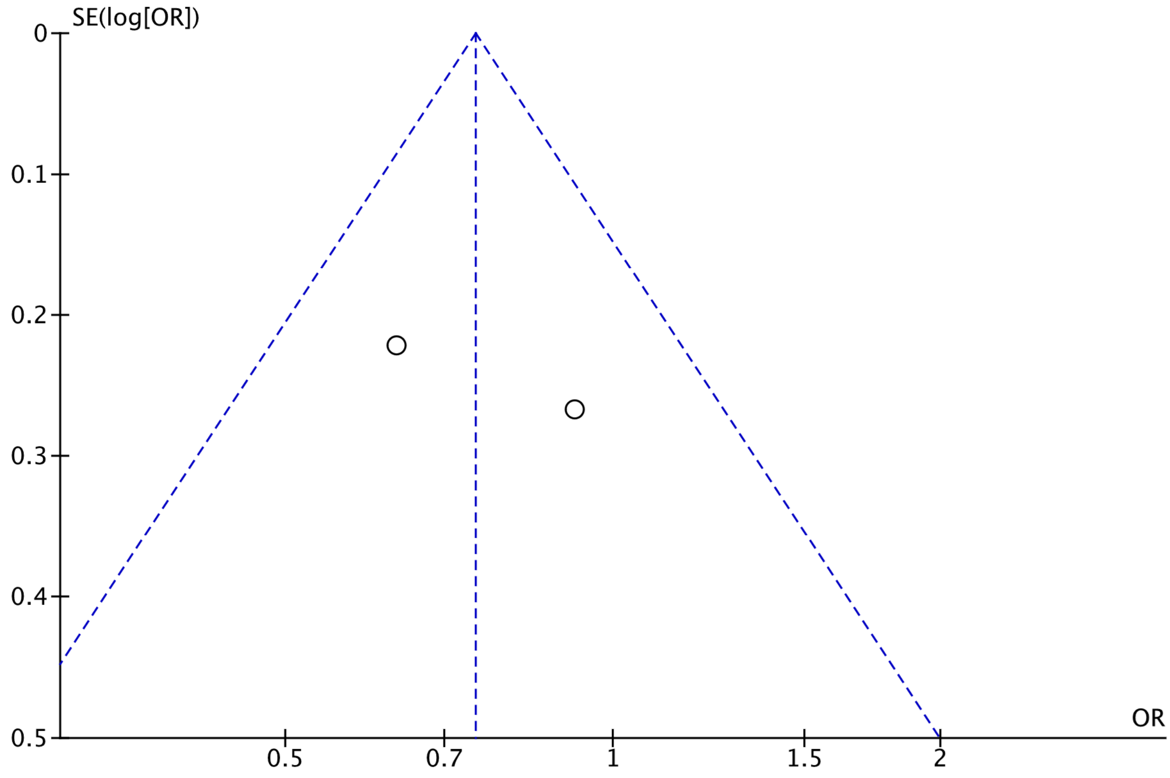

Figure S5. Funnel plot for 1 year patient survival for CIT shorter and longer than 4 hours.

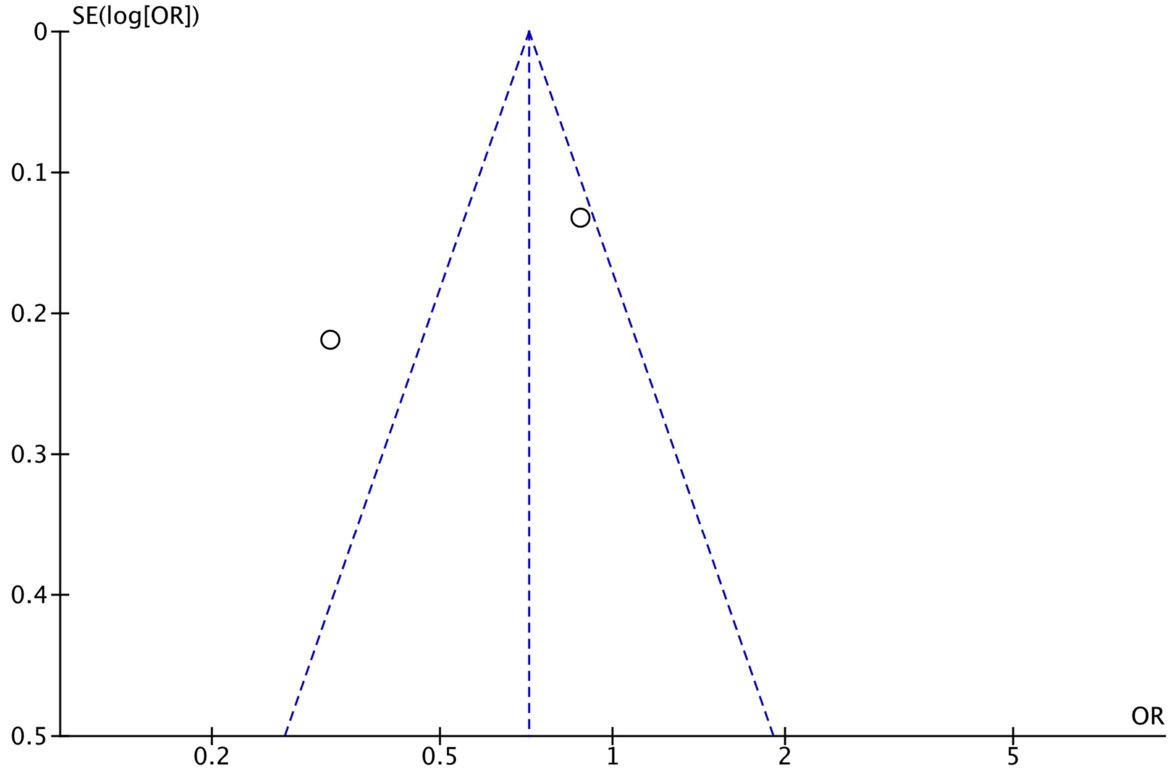

Figure S6. Funnel plot for 5-year patient survival for CIT shorter and longer than 4 hours.

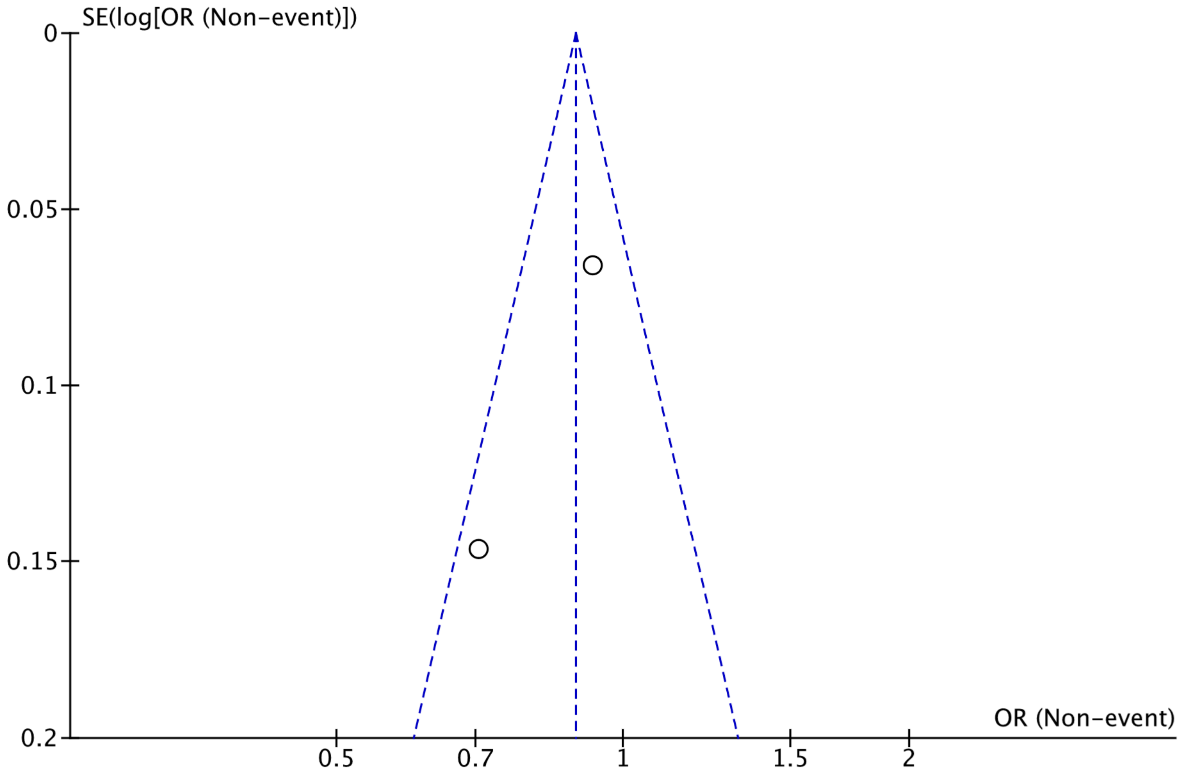

Figure S7. Funnel plot for 10-year patient survival for CIT shorter and longer than 4 hours.

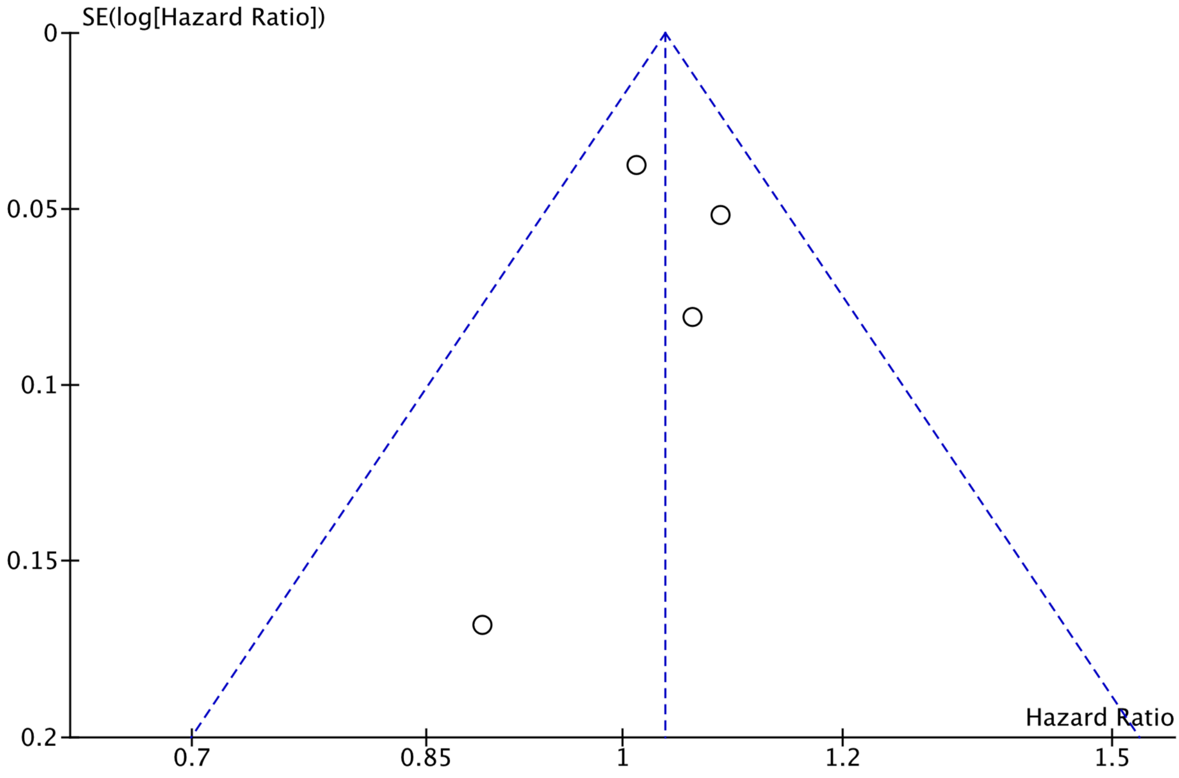

Figure S8. Funnel plot for 5- year graft survival comparing 0-2 hours versus
2-4 hours of CIT.

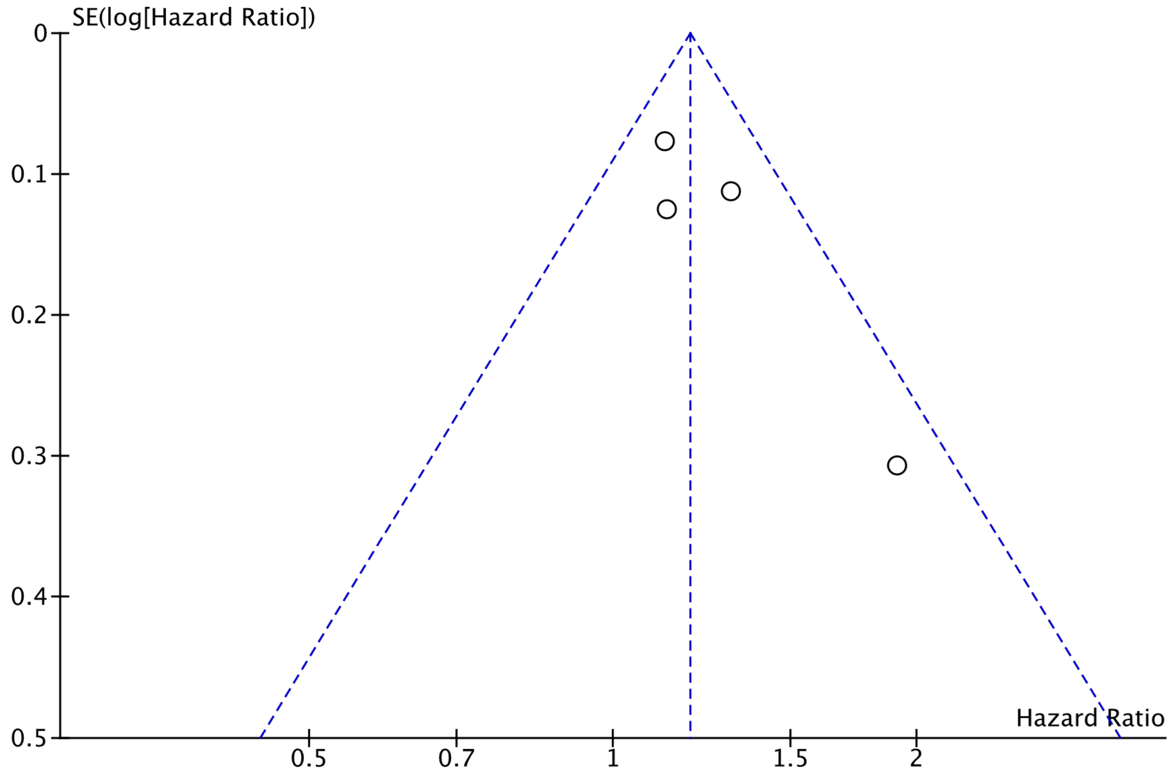

Figure S9. Funnel plot for 5- year graft survival comparing 0-2 hours versus 4-8 hours of CIT.

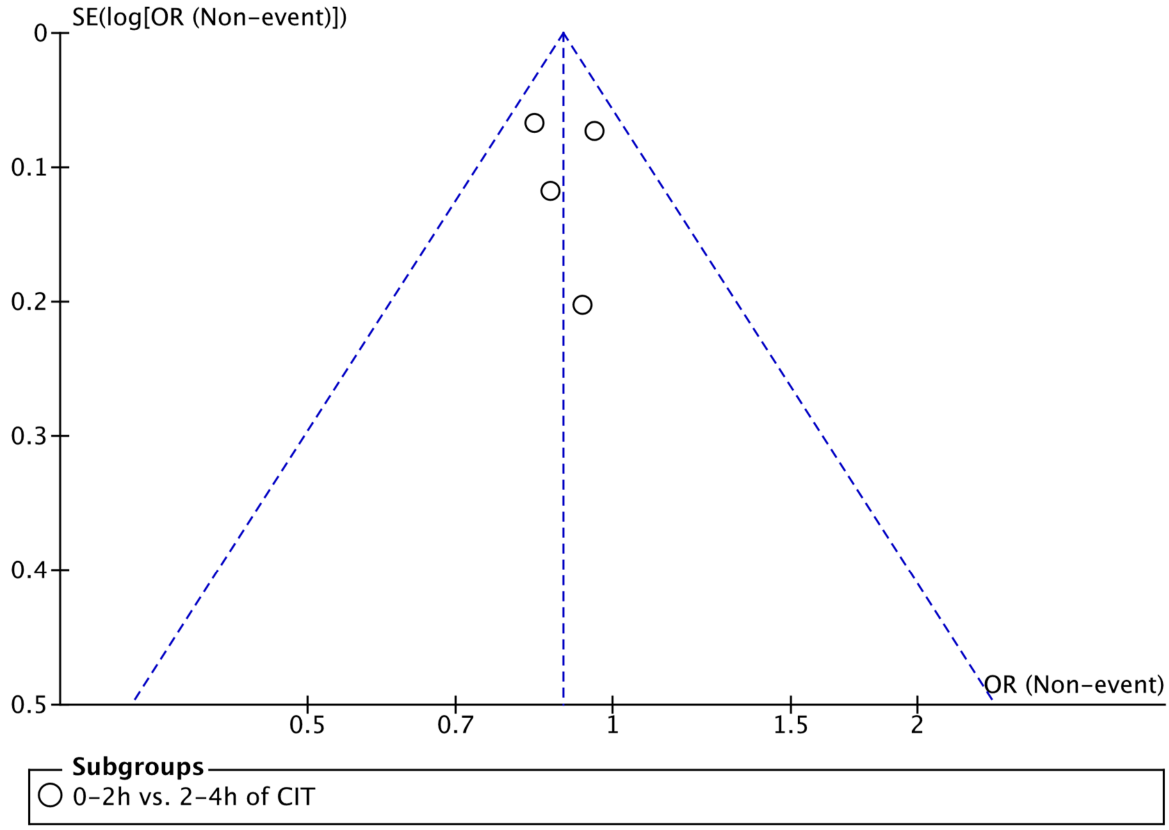

Figure S10. Funnel plot for DGF comparing 0-2 hours versus 2-4 hours of CIT.

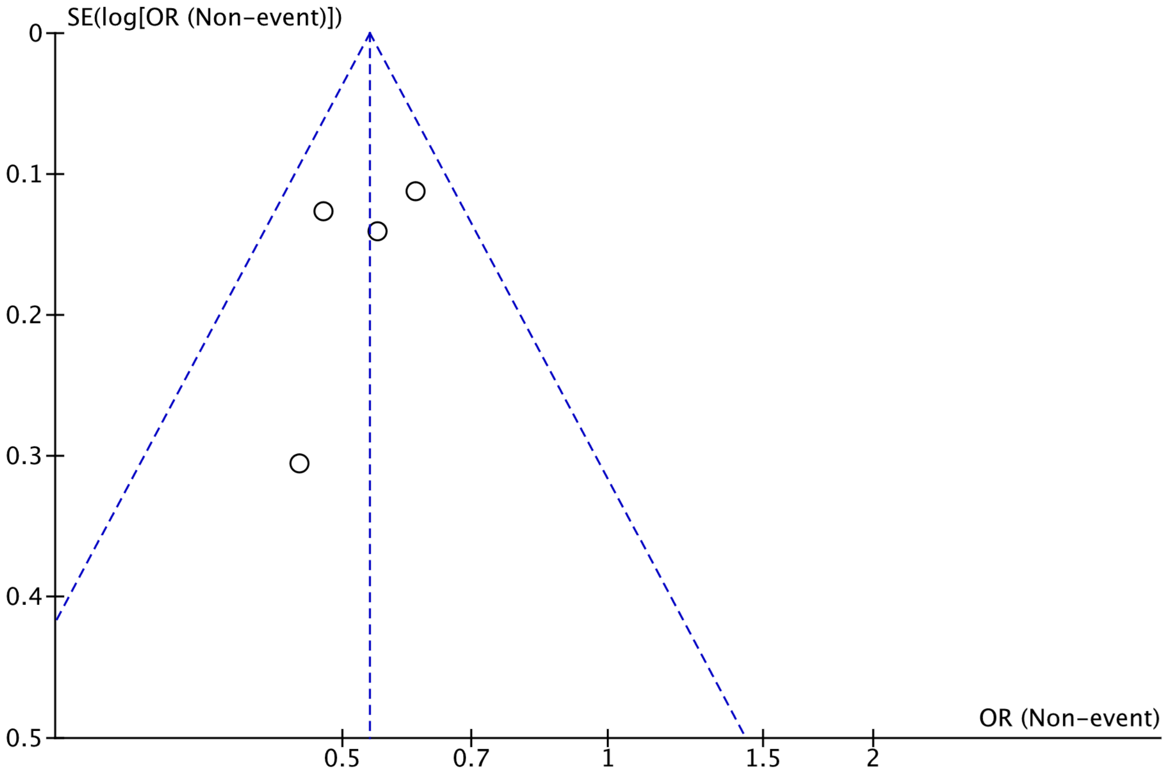

Figure S11. Funnel plot for DGF comparing 0-2 hours versus 4-6 hours of CIT.

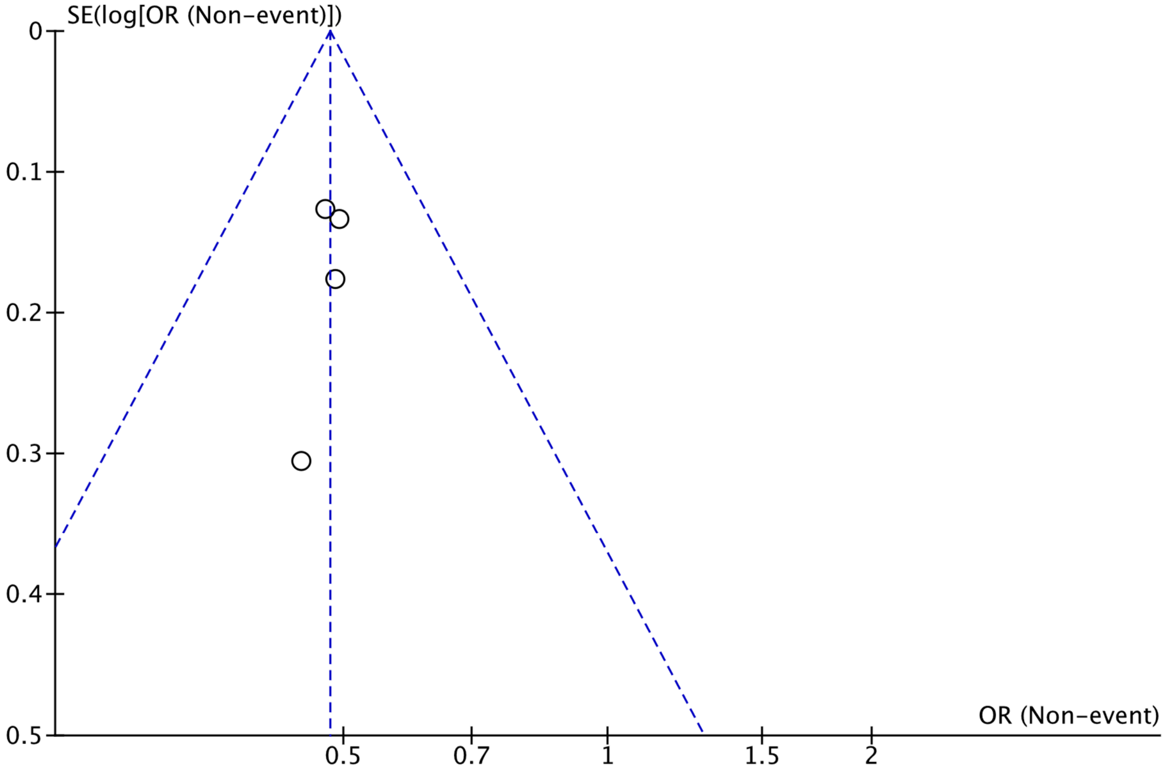

Figure S12. Funnel plot for DGF comparing 0-2 hours versus 6-8 hours of CIT.

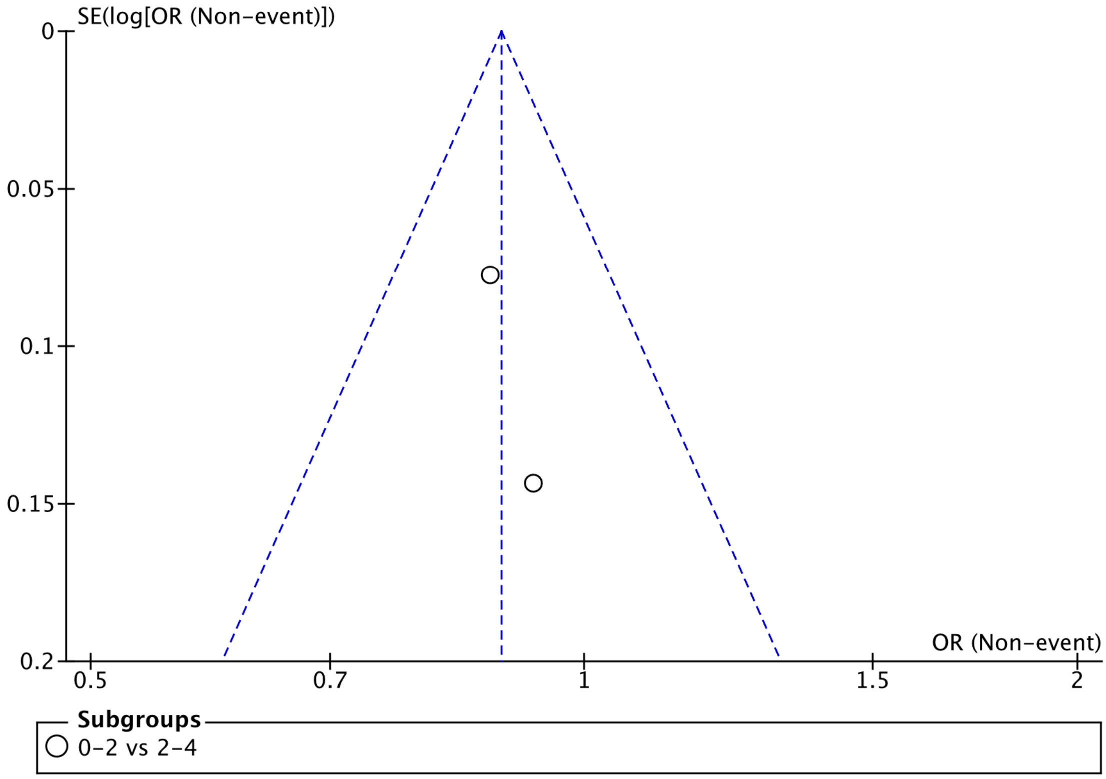

(**a**)

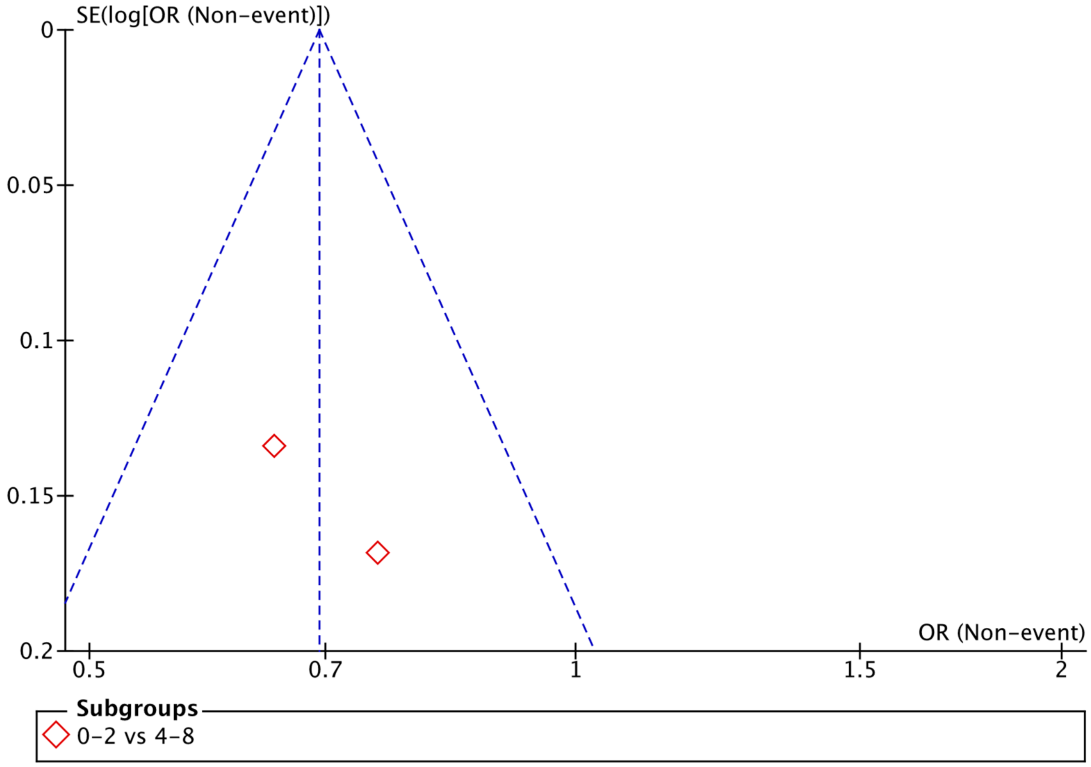

(**b**)

Figure S13. (**a**) Funnel plot for 1-year graft survival comparing 0-2 hours versus 2-4 hours of CIT. (**b**) Funnel plot for 1-year graft survival comparing 0-

Table S3: variables included in the multivariate analysis per study.

**Gill**	recipient factors (age, sex, race, ABO blood group, peak panel reactive antibody level, diabetes as cause of ESRD, dialysis duration before kidney transplantation, KPD); donor factors (age, sex, race, ABO blood group, body mass index); transplant and immunological factors (year of transplantation, HLA mismatches, warm ischemia time, induction, calcineurin inhibitors, antimetabolites, corticosteroids).
**Krishnan**	study indicates that it uses multivariate regression analysis but does not specify which variables it considers.
**Nath**	Initially, univariable analyses were performed to compare across the three cold ischaemia time (CIT) groups. To account for the ordering of the CIT groups Multivariable analyses were then performed, to account for differences in demographics across the CIT groups. Study does not further specify which variables it corrects for. Factors reported by the author as not included and might be a limitation: initial warm ischaemia time, anastomosis time, vessel anatomy and recipient body mass index have been shown to affect outcome in cadaveric kidney transplants.
**Nassiri**	Donor, recipient, and transplant characteristics, including donor and recipient age, donor and recipient gender, donor and recipient race, CIT, HLA matching, and ABO compatibility, were summarized for the entire cohort and by the categories of donor age and CIT. For both outcomes, donor age and CIT were included in the model and any other covariate significant at $P \leq .05$ from a forward selection process. Covariates entered into each model were those with $P \leq .20$ on univariate analysis for the respective outcome.
**Simpkins**	The recipient covariates that were examined included: age, gender, race/ethnicity, ABO blood group, history of diabetes mellitus, history of hypertension, history of pretransplant blood transfusion, history of previous pregnancy, peak panel-reactive antibody (PRA) level and history of pretransplant dialysis. The donor covariates included: age, gender, race/ethnicity and ABO blood group. Immunologic match was examined using separate covariates for the number of HLA-A, HLA-B and HLA-DR mismatches that were present between the donor and recipient.
